# Supplementary figures and images for: Association of preoperative body mass index with postoperative complications and survival for patients with gastric cancer: A systematic review and meta-analysis
Source: PLoS One. 2025 Jan 28;20(1):e0317985. doi: 10.1371/journal.pone.0317985 (PMC11774357; doi:10.1371/journal.pone.0317985)

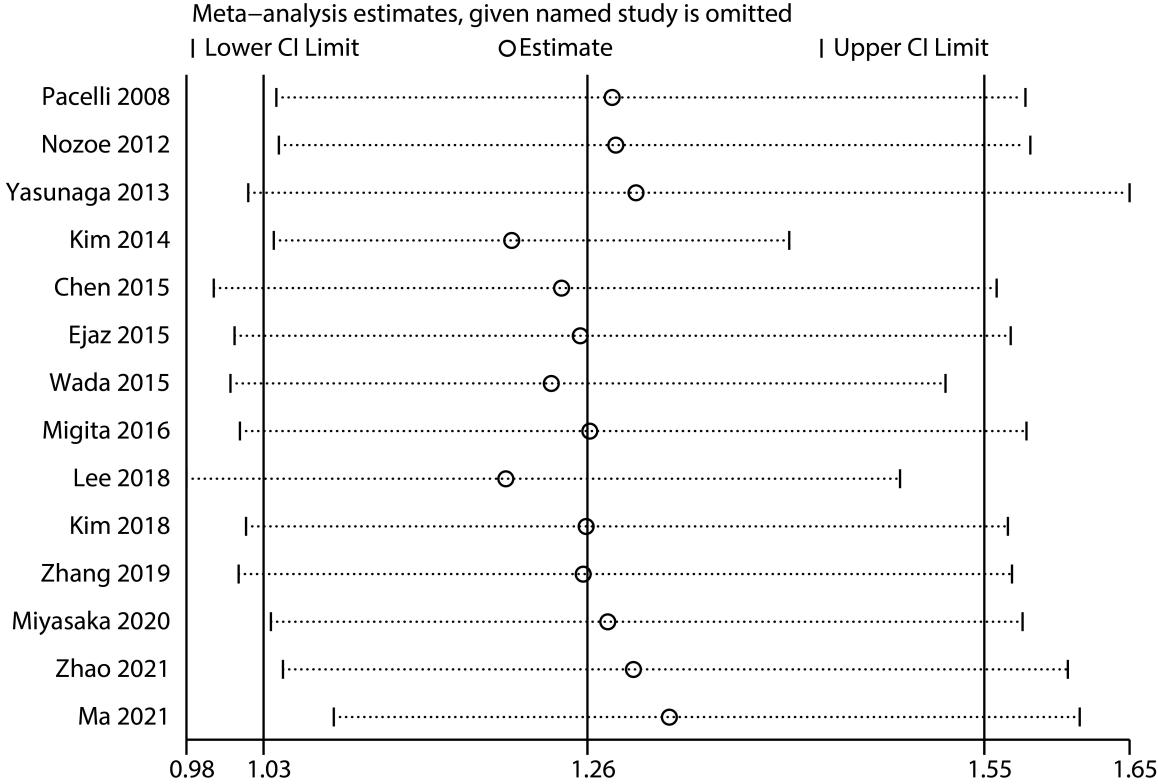

Supplement: S1 Fig — (TIF) [file pone.0317985.s002.tif]

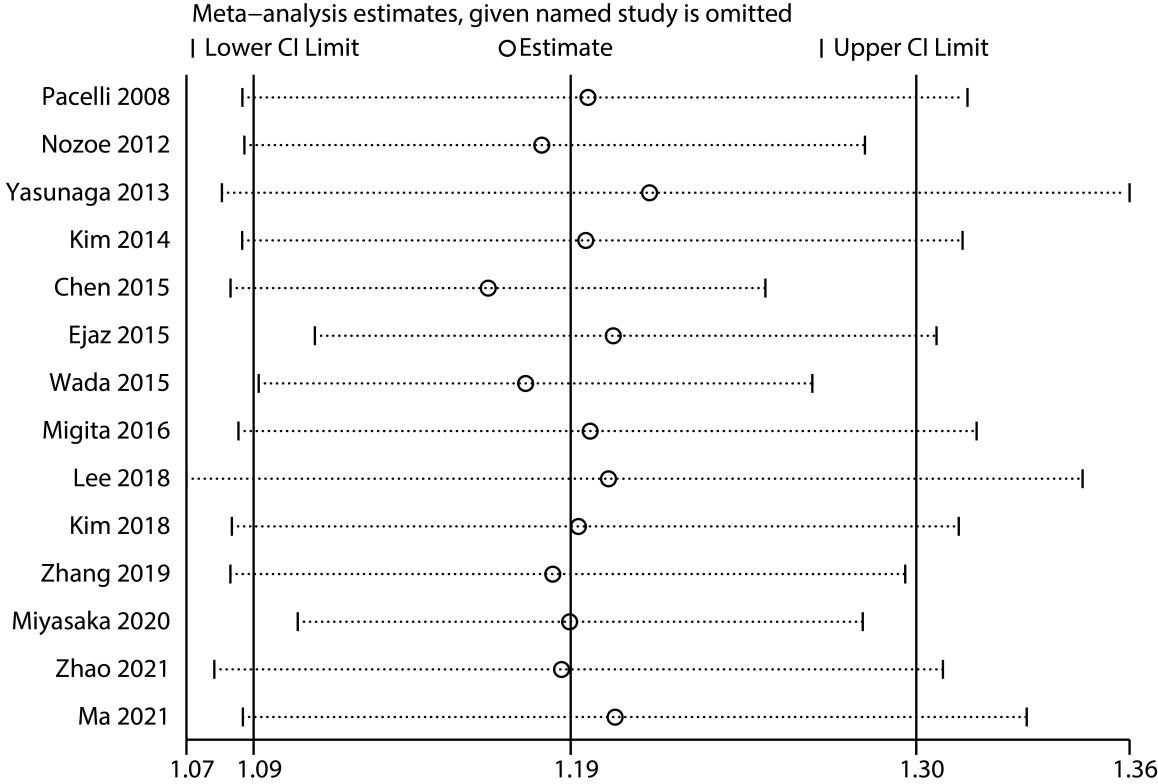

Supplement: S2 Fig — (TIF) [file pone.0317985.s003.tif]

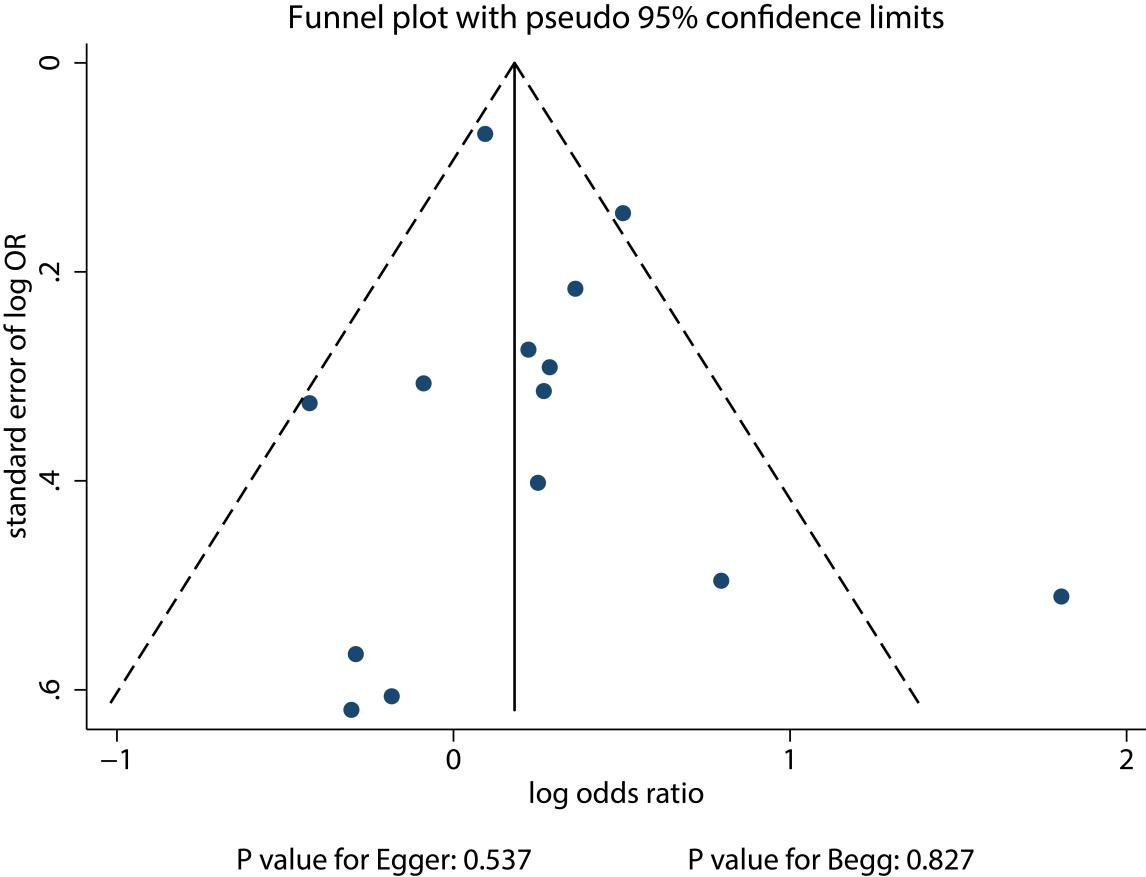

Supplement: S3 Fig — (TIF) [file pone.0317985.s004.tif]

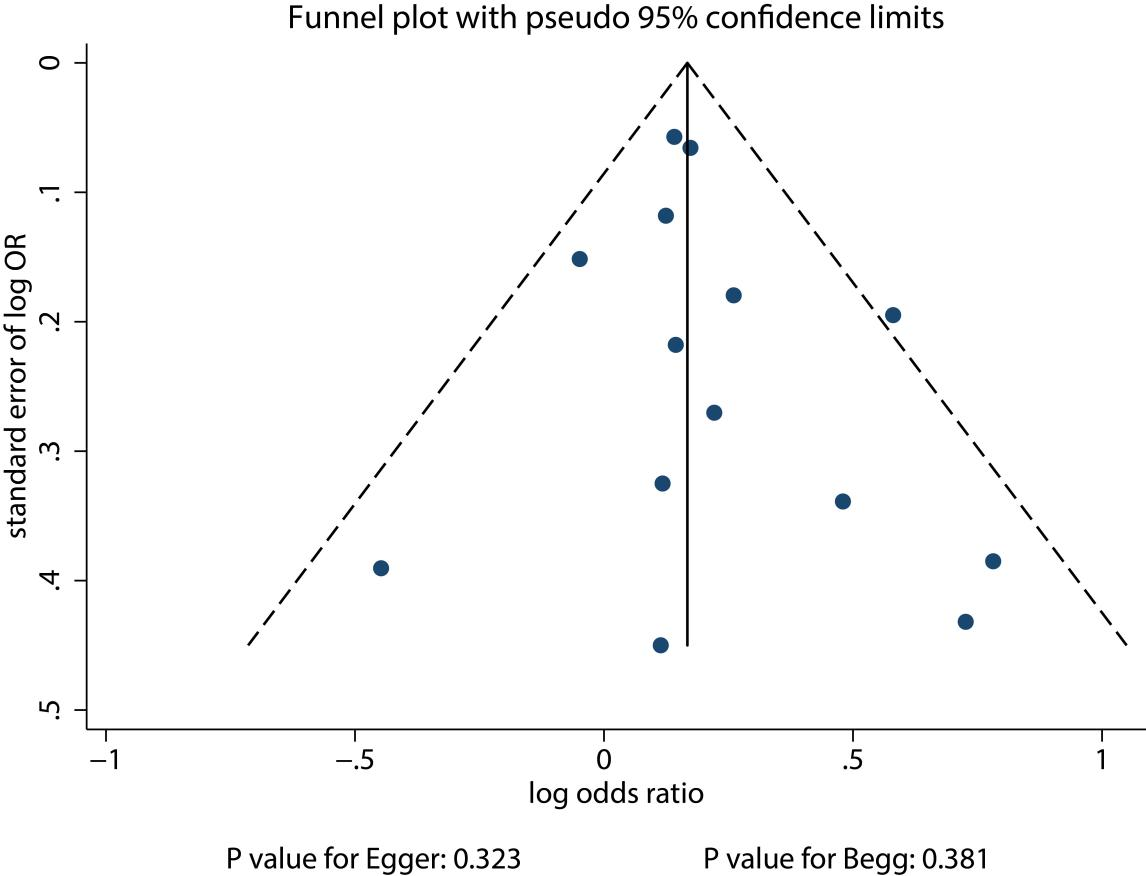

Supplement: S4 Fig — (TIF) [file pone.0317985.s005.tif]

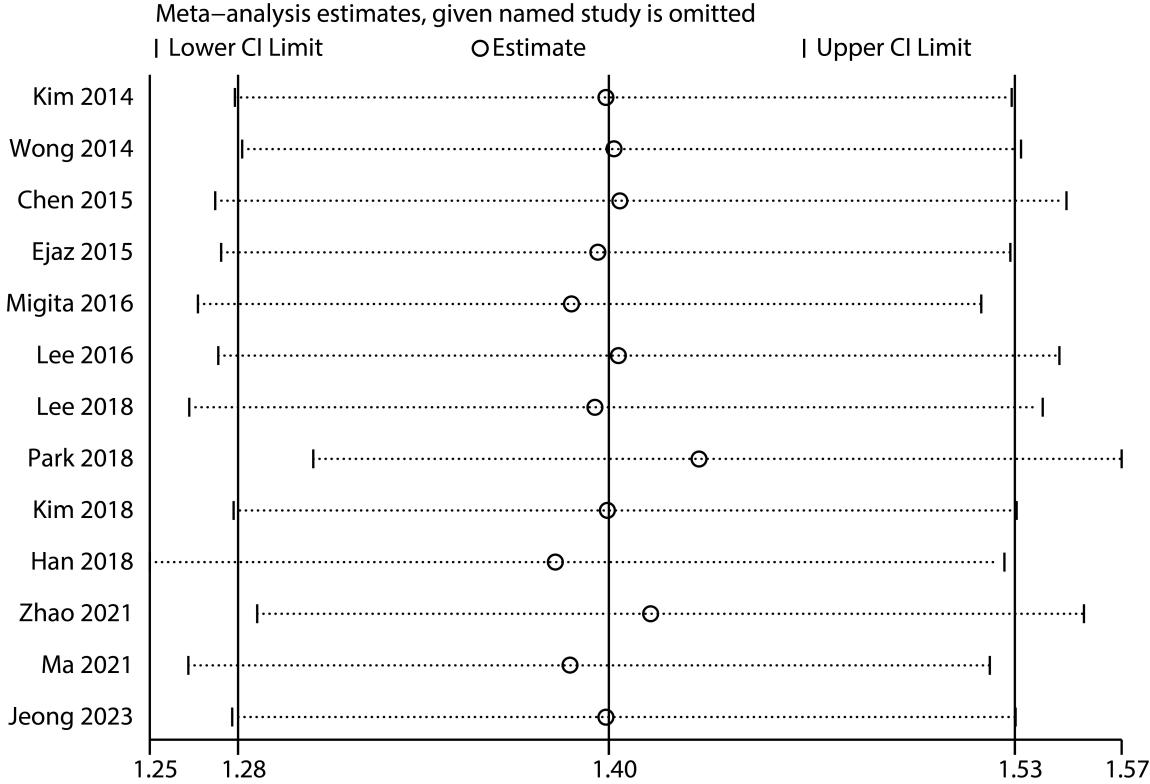

Supplement: S5 Fig — (TIF) [file pone.0317985.s006.tif]

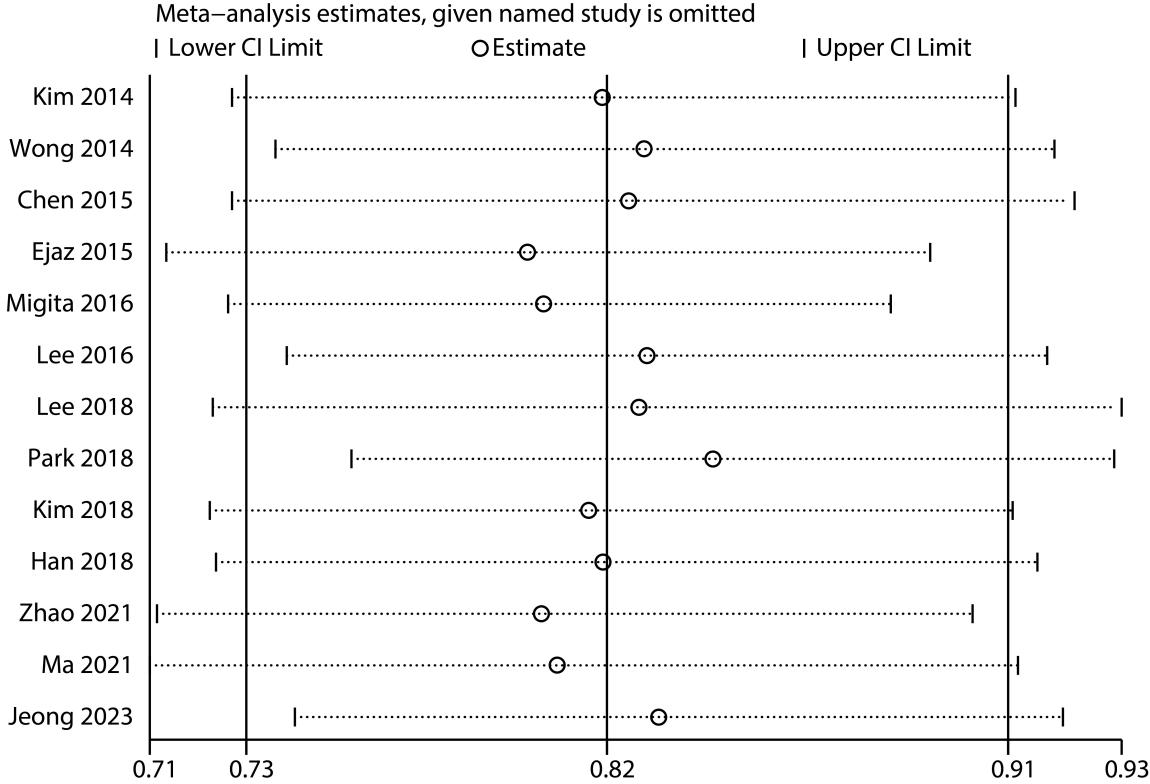

Supplement: S6 Fig — (TIF) [file pone.0317985.s007.tif]

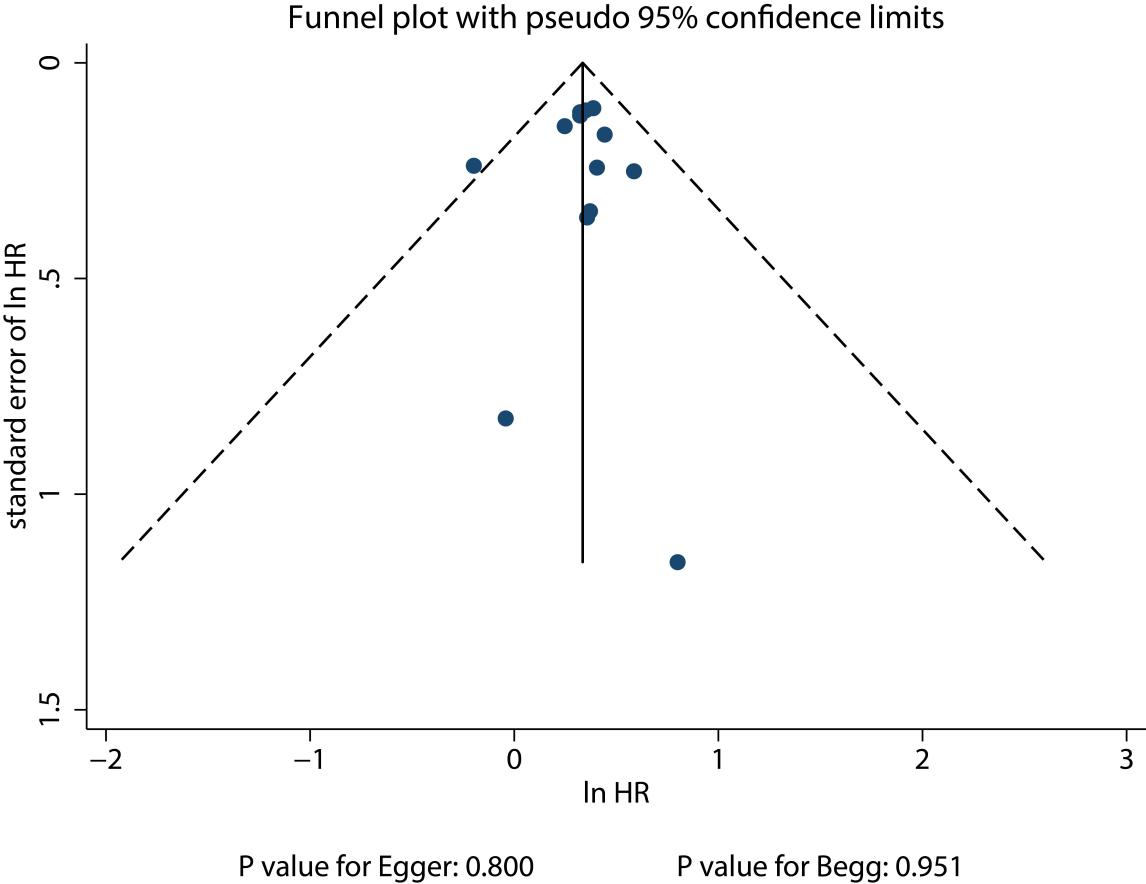

Supplement: S7 Fig — (TIF) [file pone.0317985.s008.tif]

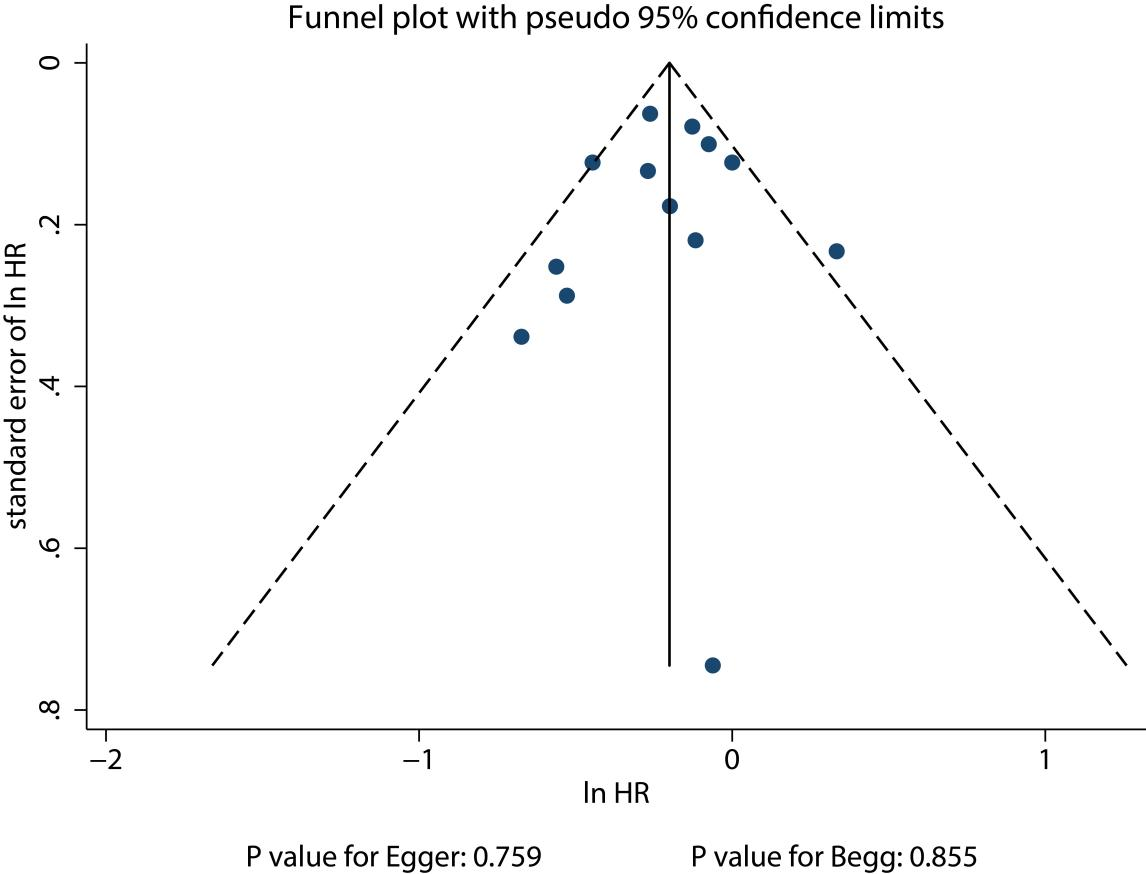

Supplement: S8 Fig — (TIF) [file pone.0317985.s009.tif]

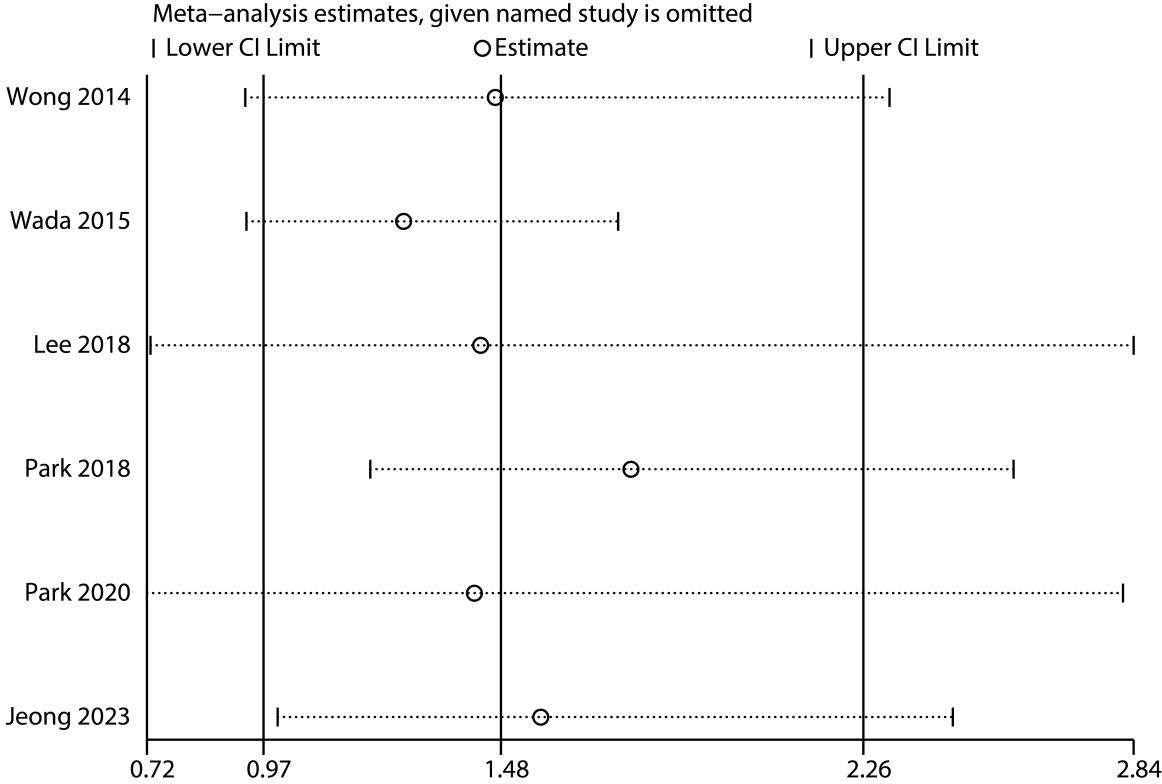

Supplement: S9 Fig — (TIF) [file pone.0317985.s010.tif]

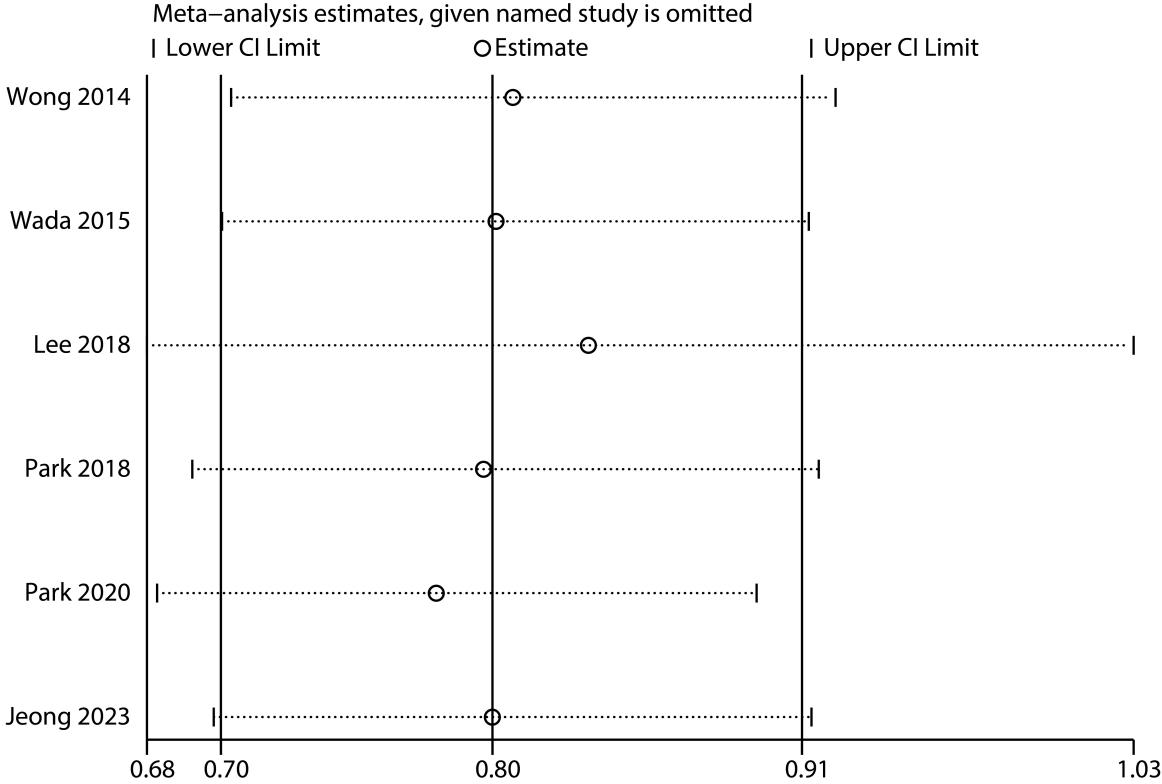

Supplement: S10 Fig — (TIF) [file pone.0317985.s011.tif]

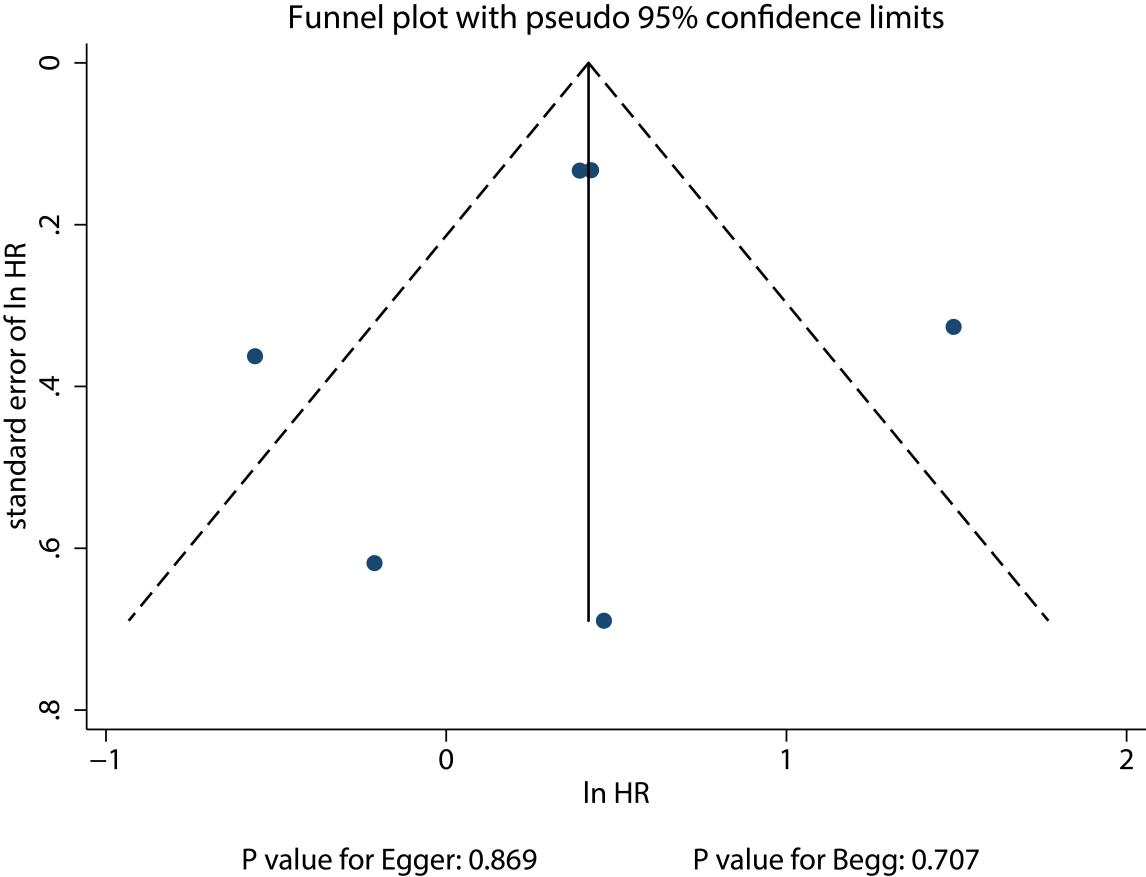

Supplement: S11 Fig — (TIF) [file pone.0317985.s012.tif]

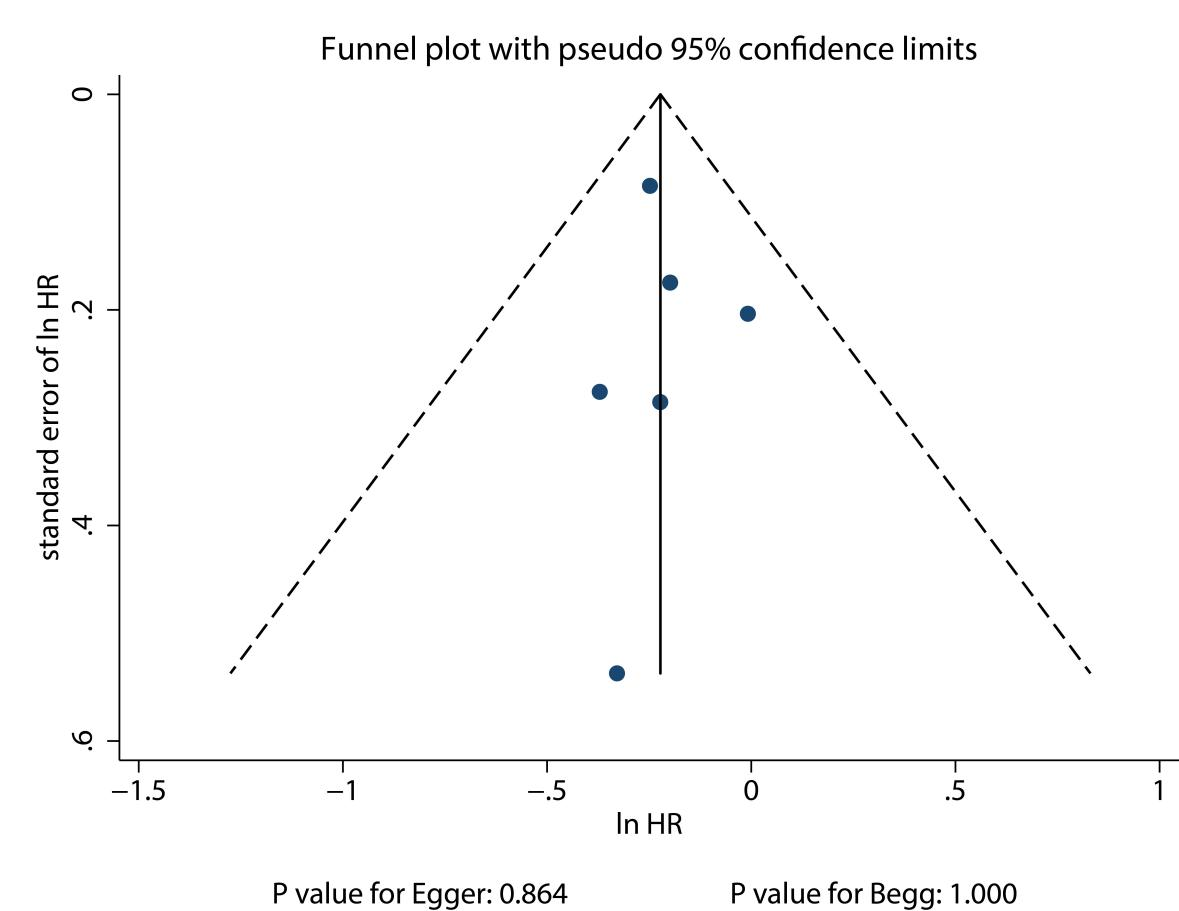

Supplement: S12 Fig — (TIF) [file pone.0317985.s013.tif]
