# Supplementary material for: Association of preoperative body mass index with postoperative complications and survival for patients with gastric cancer: A systematic review and meta-analysis
Source: PLoS One. 2025 Jan 28;20(1):e0317985. doi: 10.1371/journal.pone.0317985 (PMC11774357; doi:10.1371/journal.pone.0317985)
Supplement: S1 Table — (DOC) [file pone.0317985.s014.doc]

S1 Table. Quality scores of prospective cohort studies using Newcastle-Ottawa Scale.

| Study | Selection | | | | Comparability | Outcome | | | NOS |
| --- | --- | --- | --- | --- | --- | --- | --- | --- | --- |
|  | Representativeness of the exposed cohort | Selection of the non exposed cohort | Ascertainment  of exposure | Demonstration that outcomes was not present at start of study | Comparability on the basis of the design or analysis | Assessment of outcome | Adequate follow-up duration | Adequate follow-up rate | Overall score |
| Pacelli 2008 [34] | 0 | 1 | 1 | 1 | 1 | 1 | 0 | 1 | 6 |
| Nozoe 2012 [35] | 0 | 1 | 1 | 1 | 2 | 1 | 0 | 1 | 7 |
| Yasunaga 2013 [36] | 1 | 1 | 1 | 0 | 1 | 1 | 0 | 1 | 6 |
| Kim 2014 [37] | 1 | 1 | 1 | 1 | 1 | 1 | 0 | 1 | 7 |
| Wong 2014 [38] | 0 | 1 | 1 | 1 | 2 | 1 | 0 | 1 | 7 |
| Chen 2015 [19] | 1 | 1 | 1 | 1 | 1 | 1 | 0 | 1 | 7 |
| Ejaz 2015 [39] | 1 | 1 | 1 | 1 | 1 | 1 | 0 | 1 | 7 |
| Wada 2015 [40] | 1 | 1 | 1 | 0 | 2 | 1 | 0 | 1 | 7 |
| Migita 2016 [41] | 1 | 1 | 1 | 0 | 2 | 1 | 0 | 1 | 7 |
| Lee 2016 [42] | 1 | 1 | 1 | 0 | 1 | 1 | 0 | 1 | 6 |
| Feng 2018 [43] | 1 | 1 | 1 | 1 | 2 | 1 | 0 | 1 | 8 |
| Lee 2018 [13] | 1 | 1 | 1 | 0 | 1 | 1 | 0 | 1 | 6 |
| Park 2018 [44] | 1 | 1 | 1 | 1 | 2 | 1 | 0 | 1 | 8 |
| Kim 2018 [45] | 1 | 1 | 1 | 1 | 1 | 1 | 0 | 1 | 7 |
| Wang 2018 [46] | 1 | 1 | 1 | 1 | 1 | 1 | 0 | 1 | 7 |
| Han 2018 [47] | 1 | 1 | 1 | 0 | 1 | 1 | 0 | 1 | 6 |
| Zhang 2019 [48] | 1 | 1 | 1 | 0 | 1 | 1 | 0 | 1 | 6 |
| Park 2020 [14] | 1 | 1 | 1 | 1 | 1 | 1 | 0 | 1 | 7 |
| Miyasaka 2020 [49] | 0 | 1 | 1 | 1 | 2 | 1 | 0 | 1 | 7 |
| Zhao 2021 [17] | 1 | 1 | 1 | 1 | 1 | 1 | 0 | 1 | 7 |
| Ma 2021 [15] | 1 | 1 | 1 | 1 | 2 | 1 | 0 | 1 | 8 |
| Jeong 2023 [50] | 1 | 1 | 1 | 1 | 1 | 1 | 0 | 1 | 7 |
