# Supplementary material for: Association of preoperative body mass index with postoperative complications and survival for patients with gastric cancer: A systematic review and meta-analysis
Source: PLoS One. 2025 Jan 28;20(1):e0317985. doi: 10.1371/journal.pone.0317985 (PMC11774357; doi:10.1371/journal.pone.0317985)
Supplement: Attachment — Submitted filename: Response to reviewer.docx [file pone.0317985.s017.docx]

**Dear editor,**

Thanks for your kind reply, and thanks for the reviewers’ beneficial comments. We have revised the manuscript according to the reviewers’ comments and the point to point response is enclosed to this file. The reply was noted in RED.

**Response to reviewer 1**

**General comments:** In this article, Zhenzhen and colleagues conducted a systematic review and meta-analysis to evaluate postoperative outcomes (complications, disease-free survival, and overall survival) in individuals undergoing surgery for gastric cancer (GC) based on preoperative BMI categories (controls: BMI 18.5–25; underweight: BMI <18.5; overweight: BMI >25 kg/m²). The results show that both underweight and overweight individuals have a higher risk of postoperative complications compared to controls. Overweight individuals exhibited improved overall and disease-free survival, while underweight individuals had worse outcomes in both.
The study’s methodology is robust, and the results are straightforward. Moreover, the authors acknowledge some unavoidable limitations. However, several points merit further consideration:
Response: As behalf of all co-authors, I would like to appreciate this referee due to thoughtful comments proposed by the peer review. We have made revisions to the manuscript in accordance with the reviewer's comments.

Question 1: A revision of the manuscript’s English language by a native speaker would improve clarity, as some phrases may be misleading (e.g., "increasing evidence suggests that nutritional status is related to the postoperative incidence of different types of cancer," or "preoperative underweight may be due to excessive nutritional intake caused by tumor aggressiveness," and "male patients are more likely to develop GC than female patients, leading to poorer prognosis").
Response: Thanks for this suggestion, and these sentences have already changed in the revised manuscript, as follows: “Accumulating evidence suggests that nutritional status is associated with the prognosis of various types of cancer”; “A preoperative underweight status can result from inadequate nutritional intake, which the aggressive nature of the tumor may exacerbate”; and “male patients are more prone to develop GC than female patients, and their prognosis tends to be less favorable, primarily due to differences in hormone levels, biochemical mechanisms, and healthcare-seeking behaviors between men and women”. Moreover, The entire text has been revised by native English-speaking experts at Editage company.

Question 2: The authors observed differences in subgroup analyses between studies conducted in Eastern vs. Western countries. The European Association for the Study of Obesity (EASO) advocates for ethnicity-specific BMI cut-offs, as evidence indicates that Asian populations have distinct relationships between BMI, body fat percentage, and health risks compared to Caucasian populations. Could the observed differences be attributed to the use of a universal BMI cut-off, which overlooks these physiological differences? Commenting on this would enrich the discussion.
Response: Thanks for this suggestion. In subgroup analyses, we did not find a significant difference between Eastern and Western countries regarding the association between preoperative BMI and the prognosis of GC patients (see Table 3). Consequently, we have stated this observation in the limitations section. ***Please refer to Para 6, section Discussion, as follows:*** “Fourth, we adopted the universal BMI classification criteria; however, the impact of overweight and underweight populations on the prognosis of cancer patients differs between Eastern and Western countries. This variation influences the effect of the preoperative BMI on the prognosis of patients with GC”

Question 3: Two significant factors not considered in the study, which may have impacted the results, are tumor staging and the potential administration of chemotherapy. This is a critical limitation that should be emphasized.
Response: Thanks for this suggestion. We are aware that tumor staging and postoperative chemotherapy regimens can significantly affect patient outcomes. Nevertheless, our inability to access specific stratified data has hindered our efforts to further explore the relationship between preoperative BMI and the prognosis of GC patients, limiting our capacity for additional exploratory analyses. We have included this limitation in the respective section of our discussion. ***Please refer to Para 6, section Discussion, as follows:*** “Fifth, tumor staging and postoperative chemotherapy regimens significantly influenced the prognosis of patients with GC; however, detailed stratified data to further investigate the association between preoperative BMI and the prognosis of patients with GC were not available.”

Question 4: An intriguing finding is the association between preoperative overweight status and improved overall and disease-free survival. While the authors suggest that this may be due to early-stage cancer, it is essential to note that obesity is a known risk factor for GC. This observation should be highlighted, otherwise the results of this study could be misinterpreted.

Response: Thanks for this suggestion, and these results have already re-explanation in the revised manuscript. ***Please refer to Para 4, section Discussion, as follows:*** “A preoperative overweight status may be associated with improved OS and DFS, a phenomenon known as the “obesity paradox.” The underlying mechanism could involve overweight individuals having greater nutritional reserves and metabolic support, which aid in resisting physiological stress during treatment, as well as anti-inflammatory factors potentially produced by fat tissue, influencing the tumor microenvironment, and indirectly affecting tumor progression [61]. Nonetheless, overweight and obesity remain recognized as risk factors for GC, suggesting that they play distinct roles in the early stages of disease initiation and development [62]”

**Response to reviewer 2**

**General comments:** This meta-analysis aims to elucidate potential associations between preoperative BMI and the risk of postoperative complications, as well as survival outcomes in GC patients.

Response: As behalf of all co-authors, I would like to appreciate this referee due to thoughtful comments proposed by the peer review. We have made revisions to the manuscript in accordance with the reviewer's comments.

Question 1: The study demonstrated that both preoperative underweight and overweight statuses based on BMI were associated with an increased risk of postoperative complications. However, the BMI does not distinguish between fat mass and muscle mass, which as widely reported may influence the survival of cancer patients. Therefore, it would have been interesting to also evaluate (perhaps as subgroup analysis) the potential associations between body composition and the risk of postoperative complications or survival outcomes. Furthermore, as you mentioned in the discussion the excessive abdominal fat may affect the operating procedure and lead to increased postoperative complications. Therefore, it would have been appropriate to also consider the waist circumference in the subgroups analysis.
Response: Thanks for this suggestion. We are aware that both fat mass and muscle mass can influence patient outcomes, and furthermore, waist circumference may exhibit a significant association with the connection between preoperative BMI and the prognosis of GC patients. However, upon reviewing all included studies, we found that none of the studies provided information on fat mass, muscle mass, or waist circumference. Consequently, we have addressed this limitation in the respective section of our report. ***Please refer to Para 6, section Discussion, as follows:*** “Sixth, the association of preoperative BMI with the prognosis of patients with GC might be affected by fat mass, muscle mass, and waist circumference; however, this information was not available in the included studies.”

Question 2: In addition, the overweight and obese patients with GC may also have weight-related metabolic or cardiovascular complications that may influence the operating procedure and postoperative complications or survival outcomes, therefore it would have been suitable to consider the presence or absence of these complications in the subgroups analysis.

Response: Thanks for this suggestion. We acknowledge that GC patients who are overweight or obese may also suffer from weight-related metabolic or cardiovascular diseases, which could impact surgical procedures, postoperative complications, or survival outcomes. However, the studies included in our analysis did not provide stratified data based on these factors. Furthermore, the analyses of survival outcomes were based on adjusted results, with varying correction factors across studies, albeit a minority of them did adjust for obesity-related metabolic or cardiovascular disorders. Hence, we have highlighted this limitation within the dedicated section of our study. ***Please refer to Para 6, section Discussion, as follows:*** “Seventh, weight-related metabolic or cardiovascular complications in overweight or obese patients with GC may affect surgical procedures, postoperative complications, and survival outcomes. Nonetheless, the included studies did not provide stratified data on weight-related metabolic or cardiovascular complications”
